# Stem cell proliferation patterns as an alternative for *in vivo* prediction and discrimination of carcinogenic compounds

**DOI:** 10.1038/srep45616

**Published:** 2017-05-03

**Authors:** An-Sofie Stevens, Maxime Willems, Michelle Plusquin, Jan-Pieter Ploem, Ellen Winckelmans, Tom Artois, Karen Smeets

**Affiliations:** 1 Zoology: Biodiversity and Toxicology, Centre for Environmental Sciences, Hasselt University, Agoralaan, building D, BE 3590, Diepenbeek, Belgium; 2 Laboratory of Pharmaceutical Technology, Faculty of Pharmaceutical Sciences, Ghent University, Ottergemsesteenweg 460, BE 9000, Ghent, Belgium; 3 Laboratory of Environmental Toxicology and Aquatic Ecology, Ghent University, Jozef Plateaustraat 22, BE 9000 Ghent, Belgium; 4 MRC/PHE Centre for Environment and Health, School of Public Health, Imperial College London, St Mary’s Campus, Norfolk Place W2 1PG, London, United Kingdom; 5 Environmental biology, Centre for Environmental Sciences, Hasselt University, Agoralaan, building D, BE 3590, Diepenbeek, Belgium

## Abstract

One of the major challenges in the development of alternative carcinogenicity assays is the prediction of non-genotoxic carcinogens. The variety of non-genotoxic cancer pathways complicates the search for reliable parameters expressing their carcinogenicity. As non-genotoxic and genotoxic carcinogens have different cancer risks, the objective of this study was to develop a concept for an *in vivo* test, based on flatworm stem cell dynamics, to detect and classify carcinogenic compounds. Our methodology entails an exposure to carcinogenic compounds during the animal’s regeneration process, which revealed differences in proliferative responses between non-genotoxic and genotoxic carcinogens during the initial stages of the regeneration process. A proof of concept was obtained after an extensive study of proliferation dynamics of a genotoxic and a non-genotoxic compound. A pilot validation with a limited set of compounds showed that the proposed concept not only enabled a simple prediction of genotoxic and non-genotoxic carcinogens, but also had the power to discriminate between both. We further optimized this discrimination by combining stem cell proliferation responses with a phenotypic screening and by using specific knockdowns. In the future, more compounds will be tested to further validate and prove this concept.

A correct assessment of human cancer risks following exposures to chemicals necessitates accurate and reliable tests that estimate the carcinogenic potency of compounds. The 2-year rodent carcinogenicity bioassay is the gold standard for carcinogenic screenings of newly developed drugs and other chemical compounds, but high costs, long experimental periods and European (REACH) and US (ICCVAM Authorization Act) policies promote the search for alternative assays^1^,^2^,^3^,^4^,^5^. In addition, raising ethical concerns regarding animal use in scientific procedures led to the creation of organizations like EURL ECVAM (European Union Reference Laboratory for the Validation of Alternative Methods) and ICCVAM (Interagency Coordinating Committee on the Validation of Alternative Methods) to validate alternative approaches in accordance with the 3R’s (Replace, Reduce and Refine) Declaration, which includes the use of organisms with limited sentience such as invertebrates.

A challenge for the development of alternative test methods is that, besides an adequate estimation of carcinogenicity, compounds need to be classified according to their presumed predominant mechanism of action into genotoxic and non-genotoxic carcinogens, which entail different assessments of human cancer risk. While genotoxic carcinogens disturb the genomic integrity directly by interacting with the DNA and/or the cellular apparatus, non-genotoxic carcinogens deregulate multiple signaling pathways and, consequently, often have a different or higher threshold than genotoxic carcinogens to exert their carcinogenic effects^6^,^7^,^8^,^9^. The understanding and prediction of non-genotoxic carcinogens is substantially complicated by their compound-specific mechanisms of action^8^,^9^.

The present alternative *in vitro* and short term *in vivo* assays identify the majority of genotoxic carcinogens although improvements in the predictive capacity are still needed to diminish false-positive and -negative results^10^. 75–95% of non-carcinogens give irrelevant positive results in at least one test of the core battery genotoxicity tests (i.e. Ames-test, mouse lymphoma cell test and *in vitro* chromosome aberration test), which requires expensive and time-consuming follow-up *in vitro* and *in vivo* testing^10^. Moreover, false-positive results can lead to a needlessly discarding of potentially useful pharmaceuticals. Another major drawback is the sensitivity and reliability to detect non-genotoxic carcinogens, which represent up to 25% of Class I human carcinogens according to the International Agency for Research on Cancer (IARC)^3^,^5^,^8^,^10^,^11^,^12^. Consequently, non-genotoxic carcinogens often slip through the detection net and become wrongly classified as non-carcinogens. A better understanding of cellular and molecular events involved in non-genotoxic carcinogenesis is needed and given the extensiveness and complexity of these processes, *in vitro* detection methods may not be sophisticated enough to cover the full carcinogenicity response. A combination with alternative *in vivo* tests can contribute to these understandings.

Flatworms are promising organisms for cancer research: (1) Their remarkable regeneration capacity is based on a population of totipotent stem cells, which enables the study of carcinogen-induced stem cell responses during the regeneration or development of multiple tissues^13^,^14^. The process of regeneration potentially influences the organism’s cellular response to toxic exposures, with massive cell proliferation being a prerequisite for both regeneration and carcinogenesis^15^,^16^,^17^,^18^. (2) Their experimentally accessible stem cells enable *in vivo* studies of carcinogen-induced responses of pluripotent, adult stem cells within an entire animal. (3) The initiation and promotion stages of chemically-induced carcinogenesis are described in flatworms and underlying cancer and regeneration-related genes and signaling pathways were identified e.g. PTEN, FOXO, caspases, cyclins, (PI3K)-Akt pathway, RAS pathway, p53 pathway, MAPK pathway^19^,^20^,^21^,^22^,^23^,^24^ (cancer and regeneration share underlying molecular events^18^).

We exploited these flatworm characteristics to develop a concept for an *in vivo* assay to predict and classify carcinogenic compounds based on stem cell responses in the flatworm *Schmidtea mediterranea*. Carcinogen-induced stem cell proliferation responses were measured at various, physiologically meaningful time points during the organism’s regeneration process, and combined with a phenotypic screening. Possibilities to further subdivide each carcinogenic group according to mechanisms of action (MOA) were tested by comparing carcinogen-induced cell proliferation patterns in cancer-related gene knockdowns.

## Materials and Methods

### Test organism

Asexual strains of the freshwater planarian *Schmidtea mediterranea*^25^,^26^ were maintained in culture medium as described in *Pirotte et al.*^27^.

### Experimental design

Experiments were performed with regenerating animals (heads and tails) to accelerate initial responses to carcinogenic compounds, as this process of massive cell proliferation, migration and differentiation resembles the process of carcinogenesis. Animals were cut transversally (just above the pharynx) immediately before exposure to a genotoxic carcinogen, non-genotoxic carcinogen or non-carcinogen concentration, medium (control) or medium with DMSO (vehicle control). Exposures were performed in 6-well plates (4 ml/well). To exclude intrinsic effects of DMSO on cell proliferation, the DMSO vehicle concentration never exceeded 0.05% and interaction effects between the test compound and DMSO were investigated using a two-way ANOVA^17^.

Compound and concentration selection was based on Willems *et al*.^28^, data reported in the OECD DRP31 document^29^, IARC classifications and literature screens^10^,^29^,^30^,^31^. These compounds were selected as a starting point, and the amount of tested compounds will be enlarged in future validation studies. Based on above publications a distinction was made between genotoxic (DNA-reactive) carcinogens, non-DNA-reactive carcinogens and non-carcinogens. A genotoxic carcinogen must have shown to be positive in classical *in vitro* and *in vivo* genotoxicity assays. A non-genotoxic carcinogen is any chemical of which a non-DNA-reactive mechanism is responsible for its carcinogenic effect. Following compounds were included: the genotoxic carcinogens methyl methane sulphonate (MMS; Cas # 66-27-3; purity 99%) and 4 nitroquinoline-1-oxide (4NQO; Cas # 56-57-5; purity 98%), the non-genotoxic carcinogens methapyrilene hydrochloride (MPH; 135-23-9; analytical standard), cyclosporine A (CsA; Cas # 59865-13-3; purity ≥98.5%), chlorpromazine hydrochloride (CPZ; Cas # 69-09-0; purity ≥98%) and sodium phenobarbital (S-PB; Cas # 57-30-7) and the non-carcinogen D-mannitol (Dmann, Cas # 69-65-8), all purchased from Sigma-Aldrich (Saint Louis, MO, USA).

After comparing continuous versus intermittent (repeat-dose toxicity: 2 days of compound exposure, 3 days of recovery in culture medium) exposures and medium refreshment once versus twice a week, a continuous exposure with medium refreshment twice a week was found most suitable to obtain discriminative cellular responses (Fig. S5).

Animals were fed once a week with veal liver and starved for 1 week before stem cell proliferation measurements. Stem cell proliferation was measured at the time points indicated in the figures/tables^13^,^14^,^32^. Time points were chosen based on their physiological relevance: mitotic peaks after 1 and 3 days of regeneration, completed regeneration after +/−1 week, and fully completed regeneration and growth after a longer time period. Phenotypic effects were monitored daily with a stereo microscope. Based on general phenotypic effects, concentrations were chosen for each time point, to measure stem cell proliferation (low concentrations: category 1; high concentrations: category 2; Fig S4). RNA interference (RNAi) animals were exposed for 17 days, after which stem cell proliferation was measured.

### Mitotic activity of stem cells

Stem cells are the only proliferating cells in *S. mediterranea*^33^. Observed changes in cell proliferation can thus be directly linked to stem cell responses. The mitotic activity of stem cells was determined by immunostaining with anti-P-Histone H3 (ser10) antibody, biotin conjugate (Millipore cat number 16-189), performed as previously described^27^. The animals were examined with fluorescence microscopy performed with a Nikon Eclipse 80i. The total number of mitotic neoblasts was normalized to the body size of the animals. Before sampling, three photos were taken of each animal at the moment it stretches its body. Of these three photos, the average body size was calculated, using Image J (1.44p, National Institutes of Health, Bethesda, MD, USA), for normalisation afterwards. This method was preferred

### Phenotypic follow-up

An *in vivo* study allows the investigation of the entire organism’s response to carcinogenic compounds and, as such, makes it possible to link carcinogen-induced stem cell proliferation with the organism’s phenotype. At a specific time point (i.e. the moment the first phenotype appears), conditions were subdivided according to their phenotypic effects into three categories (Fig. S4). This was done for each compound. Category 1 represents the concentrations at that time point that induce no phenotypic effects, category 2 represents the concentrations at that time point that induce phenotypic effects, and category 3 represents lethal concentrations. Category 1 concentrations can become category 2 concentrations at another exposure time (Fig. S4c). Daily-monitored phenotypic parameters were tissue regression, lesion, blisters, bloating, pigmentation, behavior, regenerative success, body size, tissue outgrowth, flattened posture and organismal death^34^. Stem cell proliferation was quantified at the appearance of systemic effects (cat 2).

### RNA interference

The gene expression of tumor protein 53 (*p53*) and the stress-inducible heat shock protein 70 (*hsp70*) was, among others (*mcm6, pcna, CuZnsod, foxo and smedinx11*), selectively knocked down and effects on cell proliferation were studied after 17 days exposure to 50 μM MMS (genotoxic carcinogen), 10 μM MPH (non-genotoxic carcinogen) or culture medium (control). This exposure set-up was chosen on the basis of previous experiments that showed the strongest increases in cell proliferation after 17 days exposure to 50 μM MMS (S5). RNA interference was performed using double stranded RNA (dsRNA) probes, generated by an *in vitro* transcription system (T7 RibomaxTM Express RNAi System, Promega) as indicated by the manufacturer. The primer and probe details are summarized in Supplemental Fig. S7. The animals were injected with three 32 nl injections of 1 μg/μl dsRNA for 2 consecutive days in the pre-pharyngeal part of the gut using the Nanoject II (Drummond Scientific). The non-RNAi group was injected with water. At day 3, the animals were transversally cut in two and exposed to the different compounds. All RNAi approaches were validated with real time qPCR, with an inhibition between 20–50%).

### Statistical methods

Effects of time and carcinogenic exposures (including low and high concentrations of carcinogens) on the number of dividing cells were analyzed using an ANOVA model including time, exposure and the interaction between both covariates (3.1). Effects of carcinogenic exposures on the number of dividing cells were statistically analyzed using an ANOVA model including exposure (3.2). Effects of RNAi-knockdowns and carcinogenic exposures on the number of dividing cells were analyzed using an ANOVA model including RNAi, exposure and the interaction between both covariates (3.3). Residuals from the ANOVA model were inspected visually to ensure that the assumptions of constancy of variance and normality of errors were met. If the assumptions of normality were not met, a transformation of the data set was applied (Log, Square root, 1/x and e^x^). All statistical analyses were performed by using SAS version 9.2 (SAS institute, Cary, NC, USA). A p-value of less than 0.05 was considered significant. Significance levels of 0.1 level were also reported to empower observed patterns.

## Results

Stem cell proliferation is a robust and easily quantifiable parameter to study effects of toxic compounds on the flatworm stem cell system *in vivo*^17^,^35^. Stem cells respond quickly to chemically-induced stress, and their responses differ depending on the underlying toxic event. The objective of this study was to assess the potential of this parameter as a marker to predict and classify carcinogens by their predominant MOA (and cellular consequences) into genotoxic and non-genotoxic compounds.

Carcinogens affect the cell cycle, or effects are implemented when the cell cycle is activated. We artificially created a proliferative environment by inducing the regeneration process in the animals (see: experimental design in M&M). In this proliferation-promoting setting, we exposed the organisms to genotoxic and non-genotoxic carcinogens and monitored *in vivo* stem cell responses and organismal phenotypes. To further increase organismal sensitivities to specific types of carcinogenic exposures, organisms with an impaired function (knock down of cancer- and stress-related genes) were evaluated.

### Exposure during regeneration: genotoxic vs non-genotoxic

**Figure 1:**
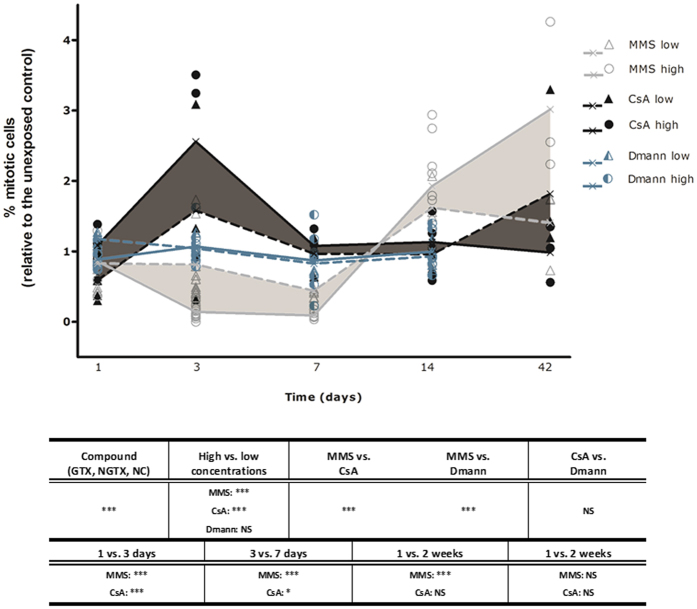
Time profile of stem cell proliferation responses following carcinogenic exposures. Mitotic divisions after 1 day (day = d), 3 days, 1 week (week = w) and 2 weeks exposure to high and low concentrations of a genotoxic carcinogen (MMS), non-genotoxic carcinogen (CsA) and non-carcinogen (Dmann). The mitotic divisions after 6 weeks exposure to MMS and CsA are also represented. The number of mitotic cells was normalized against the total body area of the worms and expressed relative to the corresponding MMS, CsA and Dmann control group per time point, respectively culture medium, culture medium with 0.05% DMSO (vehicle control) and culture medium. Average cell proliferation values at each time point are connected by lines, individual measurements are also represented (≥3 per time point). At each time point, concentrations were chosen according to the criteria described in the text and classified accordingly. Significant effects are indicated in the table: ***p < 0.01; *p < 0.1. The mean and standard errors (se) of MMS control groups are: 172.0 ± 21.9 cells/mm^2^ (1d); 368.6 ± 37.4 cells/mm^2^ (3d); 347.5 ± 8.9 cells/mm^2^ (1 w); 83.8 ± 26.0 cells/mm^2^ (2 w); 80.3 ± 9.7 cells/mm^2^ (6 w). The mean and se of CsA control groups are: 344.1 ± 42.5 cells/mm^2^ (1d); 328.3 ± 46.6 cells/mm^2^ (3d); 288.8 ± 17.7 cells/mm^2^ (1 w); 180.9 ± 15.8 cells/mm^2^ (2 w); 74.9 ± 11.6 cells/mm^2^ (6 w). The mean and se of Dmann control groups are: 352.5 ± 42.2 cells/mm^2^ (1d); 317.7 ± 26.9 cells/mm^2^ (3d); 260.6 ± 44.0 cells/mm^2^ (1 w); 135.9 ± 15.6 cells/mm^2^ (2 w). High concentrations of MMS (≤1 w: ≥200 μM; 2 w: ≥50 μM; 6 w: ≥20 μM), low concentrations of MMS (≤3d: ≤100 μM; 1 w: ≤50 μM; 2 w: ≤20 μM; 6 w: ≤1 μM), high concentrations of CsA (≤2 w: ≥0.5 μM; 6 w: ≥0.25 μM), low concentrations of CsA (≤2 w: ≤0.25 μM; 6 w: ≤0.125 μM), high concentrations of Dmann (≤3d: ≥27.4 mM; ≥1 w: ≥16.5 mM), low concentrations of Dmann (≤3d: ≤16.5 mM; ≥1 w: ≤5.5 mM). *MMS* methyl methane sulphonate, *CsA* cyclosporine A, *Dmann* d-mannitol

Stem cell proliferation was monitored at physiological meaningful time points after exposure to both low and high concentrations of a genotoxic carcinogen, a non-genotoxic carcinogen and a non-carcinogen (Fig. 1, see M&M for more details on experimental set-up). Concentrations were determined based on initial lethality screens. The kinetic proliferation profiles of the genotoxic compound MMS and the non-genotoxic compound CsA were significantly distinguishable from each other (p < 0.01) (patterns were based on different concentrations per time point, see M&M for more details on experimental set-up). The largest differences between MMS and CsA were detected before the full achievement of homeostasis (up until 2 weeks exposure; Fig. 1 and Figs S1 and S6), with notable changes between 1 and 3 days, i.e. a significant decrease for the genotoxic carcinogen MMS (p < 0.01) compared to a significant increase for the non-genotoxic carcinogen CsA (p < 0.01). The addition of the non-carcinogen Dmann into the statistical analysis only revealed significant differences with MMS exposure (p < 0.01). Concentration–dependent effects were detected for both groups of carcinogenic compounds, but not for the non-carcinogen Dmann. The stability of the measurements is represented in Fig. S6.

**Figure 2:**
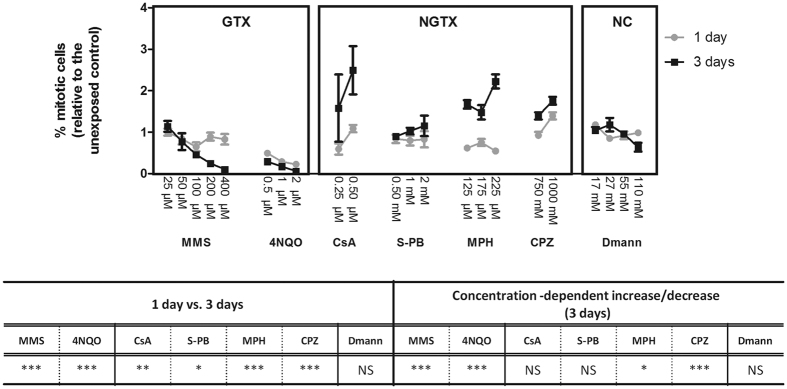
Comparison of stem cell proliferation responses after 1 and 3 days of carcinogenic exposure. Mitotic divisions after 1 and 3 days (day = d) exposure to genotoxic carcinogens (MMS, 4NQO), non-genotoxic carcinogens (CsA, S-PB) and non-carcinogens (Dmann). The number of mitotic cells was normalized against the total body area of the worms and expressed relative to the corresponding MMS, 4NQO, CsA, S-PB and Dmann control group per time point, which was culture medium for all compounds except for CsA, where culture medium with 0.05% DMSO (vehicle control) was used. Cell proliferation values of each concentration are connected by lines and are the average and standard error (se) of minimum 3 biological repeats. Significant effects are indicated in the table: ***p < 0.01; **p < 0.05; *p < 0. 1. The mean and se of MMS control groups are: 172.0 ± 21.9 cells/mm^2^ (1d) and 368.6 ± 37.4 cells/mm^2^ (3d). The average and se of 4NQO control groups are: 293.5 ± 21.6 cells/mm^2^ (1d) and 452.7 ± 30.3 cells/mm^2^ (3d). The average and se of CsA control groups are: 344.1 ± 42.5 cells/mm^2^ (1d) and 328.3 ± 46.6 cells/mm^2^ (3d). The average and se of S-PB control groups are: 289.6 ± 41.1 cells/mm^2^ (1d) and 424.5 ± 10.9 cells/mm^2^ (3d). The average and se of Dmann control groups are: 352.5 ± 42.2 cells/mm^2^ (1d) and 317.7 ± 26.9 cells/mm^2^ (3d). *MMS* methyl methane sulphonate, *4NQO* 4-nitroquinoline-1-oxide, *CsA* cyclosporine A, *S-PB* sodium phenobarbital, *Dmann* d-mannitol

To assess if the observed differences in cell proliferation after 1 and 3 days exposure are not compound-specific responses, and as such can potentially be generalized to other genotoxic and non-genotoxic carcinogens, we exposed the organisms to one additional genotoxic (4NQO) and 3 additional non-genotoxic (S-PB, CsA, MPH and CPZ) carcinogens (Fig. 2). More non-genotoxic carcinogens were included, as most other current tests have a limited sensitivity in detecting these compounds. By focusing on 1 and 3 days exposure, we attempt to increase the differential power between genotoxic carcinogen, non-genotoxic carcinogen and non-carcinogen-induced stem cell proliferation. Proliferation significantly differed between 1 and 3 days exposure for the genotoxic carcinogens MMS (p < 0.01) and 4NQO (p < 0.01) and for the non-genotoxic carcinogens CsA (p < 0.05) and S-PB (p < 0.1). No significant time differences were observed between 1 and 3 days of Dmann exposures. Two striking results in function of the assay were (1) the significant decrease in cell proliferation with increasing concentrations of genotoxic carcinogens after 3 days exposure (p < 0.01) compared to the concentration-dependent increase of non-genotoxic carcinogens and (2) the stronger inhibition/induction of cell proliferation after 3 days exposure to respectively genotoxic and non-genotoxic carcinogens, as compared to a 1-day-exposure.

Because regional signals in the planarian body can guide stem cell fate in the presence of DNA instability^36^, all measurements were performed per body part (head or tail parts, Fig. S3). In general, the most striking effects were detected in the tails. We additionally checked if we could specify mitotic changes per region, or if cell proliferation patterns differed along the body axis. Only for the genotoxic exposure, we sometimes observed the strongest effects in the blastema region, although this was not consistent for all samples (Fig. S2).

### Phenotypic effects and associated stem cell responses: genotoxic vs non-genotoxic exposure

**Figure 3:**
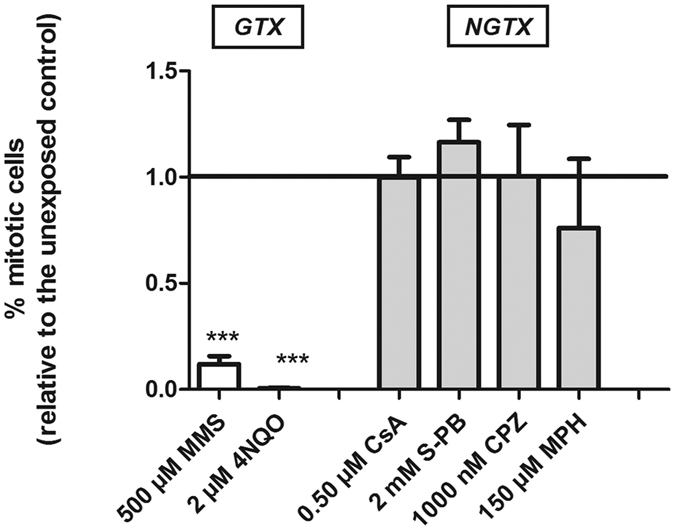
Stem cell activity responses at the appearance of phenotypic effects. Stem cell proliferation responses at the appearance of phenotypic effects (cat 2) following genotoxic (MMS and 4NQO) and non-genotoxic (S-PB, CPZ and MPH) carcinogen exposures. The number of mitotic cells was normalized against the total body area of the worms and expressed relative to the corresponding non-exposed group. The values indicated in the graphs are the mean and se of min. 3 biological repeats, except for S-PB and CPZ because of severe lysis of the animals. Significant effects as compared to the corresponding control group are indicated with stars: ***p < 0.01. Exposure times were 3 days (MMS), 1 week (4NQO) and 2 weeks (S-PB, CPZ and MPH). The mean and se of groups exposed to culture medium are: 227.7 ± 26.7 cells/mm^2^ (3 days); 212.0 ± 4.7 cells/mm^2^ (1 w); 130.9 ± 10.2 cells/mm^2^ (2 w). *MMS* methyl methane sulphonate, *4NQO* 4-nitroquinoline-1-oxide, *S-PB* sodium phenobarbital, *CPZ* chlorpromazine hydrochloride, *MPH* methapyrilene hydrochloride

To further distinguish between carcinogenic and non-carcinogenic exposures and between genotoxic and non-genotoxic carcinogens, we evaluated stem cell dynamics in relation to the induced phenotypic effects. At a specific time point (i.e. the moment of a clear phenotype induced by the compound under investigation), exposure concentrations were subdivided into categories, in which category 1 consisted of concentrations that induced no phenotypic effects, category 2 consisted of concentrations that induced phenotypic effects but no lethality at the measured time points and category 3 were lethal concentrations at that time point (Fig. S4; see also material and methods for more details). In contrast to the carcinogenic exposures, no phenotypic effects were induced by the non-carcinogen Dmann. Category 2 concentrations of genotoxic carcinogens induced phenotypic effects such as aberrant pigmentation spots, severe tissue regression, regeneration failures and aberrant behavior (Fig. S4). The strong phenotypes (tissue regression, …) eventually resulted in organismal death (at later time points), organisms with the regeneration – and pigmentation – dependent phenotypes survived during a longer experimental period. An exposure to category 2 concentrations of non-genotoxic carcinogens elicited physiological responses, which a smaller impact on the organism’s viability, e.g. changes in body size or length or an aberrant behavior. As phenotypic effects are sometimes difficult to categorize, we further enhanced the discriminative power by the quantification of cell proliferation at the appearance of phenotypic effects: Genotoxic carcinogen-induced phenotypic effects (category 2 concentrations) were associated with a significant drop in stem cell proliferation compared to non-exposed animals, while no proliferation changes were observed at the manifestation of non-genotoxic carcinogen-induced phenotypes (Fig. 3). It is possible that the strong decrease in cell proliferation of the genotoxic compounds is due to the severity of the exposure, as for this type of exposure there is often no obvious ‘buffer time’ between phenotype appearances and lethality. But also in the organisms with the less severe (and non-lethal) phenotypes due to genotoxic exposure (4NQO and MMS at later time points), we again detected strong mitotic decreases. These phenotypes showed regeneration and pigmentation disturbances. For MMS, only after an exposure time of 14 days, proliferation started to increase (Fig. 1).

### Cancer-related gene knockdowns

To increase accuracy and to provide mechanistic information, we evaluated the additional value of using knock-down animals in the assay. A more sensitive animal, or mechanism-based subdivision of carcinogenic compounds was aimed by combining carcinogenic exposures with a knockdown of stress- and cancer-related genes (*p53, hsp70, mcm6, pcna, CuZnSOD, smedinx-11, foxo*). Out of a first preliminary screen, the most divergent responses between both carcinogenic groups were obtained after a *p53* knockdown (Table 1). A knockdown of *p53* in combination with exposure to the genotoxic carcinogen (MMS) eliminated the increase in cell proliferation observed in non-RNAi, MMS-exposed animals. On the contrary, an exposure of *p53* knockdowns to a non-genotoxic carcinogen (MPH) had no additional effects on proliferative patterns in comparison with unexposed *p53* knockdowns (Table 1). *Hsp70* is used as an example of a gene knockdown which had no differential effects on both groups (Table 1).

## Discussion

Genotoxic and non-genotoxic carcinogens stimulate (uncontrolled) cell proliferation via different pathways. While genotoxic carcinogens directly induce DNA damage, non-genotoxic carcinogens indirectly act through, among others, persistent overstimulation of cell replication, induction of oxidative stress or mitogenic responses following cytotoxic effects^6^,^37^. Risks and risk assessments differ between both carcinogenic groups making their accurate prediction and discrimination essential for human health protection. In the current alternative *in vitro* tests, non-genotoxic carcinogens often slip through the detection net and become wrongly classified as non-carcinogens. Moreover, the existing tests do not categorize carcinogens into genotoxic or non-genotoxic, which is a drawback for further risk assessment.

We propose a concept based on the *in vivo* stem cell proliferation dynamics of the regenerating flatworm *Schmidtea mediterranea*, in which both the detection as well as discrimination of non-genotoxic carcinogens was realized for the tested compounds. Using a regenerative animal, we hypothesized stronger effects while exposing the animal during the physiological activation of cell proliferation^15^,^38^,^39^,^40^. This proliferative environment was created by cutting the animal in two pieces to induce regeneration. Another innovative aspect of our approach involves a comparison during a significant part of the animals’ life span, as recommended by regulatory guidelines and carcinogenicity study protocols^41^.

The time intervals exposing the strongest differences when comparing genotoxic (MMS) and non-genotoxic (CsA) exposures occurred before homeostasis, i.e. between 1 and 3 days of regeneration (around this time points, mitotic cells reach their minimum and maximum^32^) and between 1 and 2 weeks of regeneration (in between which the regeneration process is completed^42^) (Fig. 1). The proliferative inhibiting effects of MMS-induced DNA damage (and corresponding cell cycle arrest or delay) were enlarged around 3 days of exposure, when cells normally reach a regeneration-associated cell proliferation peak^9^,^43^,^44^,^45^,^46^. Also the non-genotoxic mode of action of CsA was more pronounced when cell proliferation was already induced^9^,^37^, and its proliferation-stimulating abilities even exceeded regeneration-induced peaks. The adequacy of the 1 and 3 days-exposure patterns was verified using additional genotoxic and non-genotoxic compounds: 4NQO (GTX), MPH (NGTX), CPZ (NGTX) and S-PB (NGTX), which showed similar time as well as concentration profiles (Fig. 2). More non-genotoxic carcinogens were included, as most current tests fail (or have a limited sensitivity) in detecting these compounds. For this set of compounds, we can conclude that genotoxic carcinogens were characterized by significantly fewer mitotic cells after 3 days exposure in comparison with a 1-day exposure set-up (or compared to the non-exposed control), while, on the contrary, non-genotoxic carcinogens were characterized by significantly more mitotic cells after 3 days exposure in comparison with a 1-day exposure set-up (or compared to the non-exposed control at d3). Concentration profiles at three days of regeneration increased predictive power to detect genotoxic activity. Out of these data, we propose a statistically empowered parameter-analyses between 1 and 3 days, which can easily be enrolled in high throughput screenings because of the short exposure period. We acknowledge that these findings are based on a limited set of compounds, further validation and valorization steps are needed to confirm our concept. If necessary, more detection parameters can easily be added to increase detection power without interfering with the simple set-up.

For example, a combination can be made with other time points, or concentration effects can be included. The MMS-induced cell proliferation inhibition disappeared between one and two weeks of regeneration, and again increased concentration-dependently at later time points (up until 6 weeks of exposure), while the non-genotoxic carcinogen (CsA) did no longer affect cell proliferation during this period. Proliferation patterns during an exposure interval of 1–2 weeks were also discriminative for other genotoxic (4NQO) and non-genotoxic (MPH, CsA and CPZ) carcinogens (Fig. S1). We earlier demonstrated that the 2-week exposure time point was predictive for carcinogenic compounds in the *in vivo* adult stem cell proliferation *M. lignano* assay^28^, and a combination with this test can be made to increase sensitivity.

To strengthen the discriminative power of our work-flow, we exploited its *in vivo* design by linking carcinogen-induced stem cell dynamics with organismal phenotypes (Fig. 3). A phenotypic screening offers the advantage of being cheap and fast, requiring only a stereo microscope to perform high-throughput analyses of carcinogen-induced phenotypes. The tested non-carcinogen induced no phenotypic effects and could easily be distinguished from carcinogenic compounds. The non-genotoxic carcinogens induced physiological responses that did not seem to have a direct influence on the organism’s viability, such as changes in body size or length or decreased motor coordination. The genotoxic carcinogen exposures, on the other hand, directly induced strong phenotypes indicating cell death or cell depletion, comparable to the induction of apoptosis and necrosis in other organisms^37^. At lower exposure concentrations, they showed pigmentation defects as well as regenerative failure. Systemic disorders differed between both classes of carcinogens, but discrimination is often difficult, especially for a non-specialist. Nevertheless, their moment of appearance was considered promising for quantifying stem cell proliferation. Indeed, large differences between genotoxic (MMS and 4NQO) and non-genotoxic (S-PB, CPZ and MPH) carcinogen-induced cell proliferation were recorded when phenotypic alterations appeared. Genotoxic carcinogen-induced phenotypes were correlated with major cell division blocks, explaining the observed regenerative failures and tissue regression. This link was observed for both severe and less severe genotoxic carcinogen-induced phenotypes (Fig. S4), but there was no correlation between non-genotoxic carcinogen-induced phenotypes and cellular mitosis. This can be due to the complexity of non-genotoxic carcinogens, disturbing a diversity of other vital cellular functions. A quantification of proliferative responses at the appearance of systemic effects overcomes the difficulty of defining an exposure time at which both genotoxic and non-genotoxic carcinogens, with different or higher threshold levels to exert their carcinogenic effects, can be detected^6^,^7^,^8^,^9^. In addition, there is no need to perform extensive pre-screenings to define EC10 or IC10 concentrations. Because of all of the above-cited arguments, measuring stem cell proliferation at the moment of phenotype appearance was added as a second step of the assay, to verify the results of the 1 and 3 day measurements. However, we recognize that we only used one non-carcinogen in this proof of concept. It is possible that other non-carcinogen toxicants will also induce phenotypic effects. Nevertheless, our concept can easily be combined with earlier described planarian parameters and assays to detect for f.e. neurotoxicity or behavior^47^,^48^.

A final advantage of the concept we propose here is the possibility to subdivide each group of carcinogens into additional, mechanism-based classes; or to create a more sensitive animal. In doing so, it also reconfirmed the classification of genotoxic and non-genotoxic carcinogens. Instead of transcriptional profiling and pathway analysis, we simply knocked down the genes with different functions and/or importance in the corresponding modes of action (Table 1). Out of several genes (genes that blast to *mcm6, pcna, CuZnsod, foxo, p53, hsp70 and smedinx11*), *Smed-p53* knockdowns differentially altered genotoxic and non-genotoxic proliferation profiles (Table 1). More specifically, knocking down *p53* affected the induction of cell proliferation, observed after 17 days exposure to the genotoxic carcinogen (MMS), while non-genotoxic carcinogen (MPH)-induced cell proliferation patterns were not altered. The *p53* tumor suppressor gene is a frequently used target in genetically modified rodents to increase the sensitivity to carcinogens, shorten the required bioassay time and consume fewer animals, while maintaining a high specificity^49^,^50^. In our assay, a *p53* knockdown did not only increase the organism’s sensitivity to genotoxic carcinogens, it also further discriminated genotoxic from non-genotoxic carcinogen exposures. An underlying cause of the p53-observed differences could be a dissimilarity in *p53*-driven repair systems. While *p53* can be induced by both genotoxic (DNA) and non-genotoxic (oxidative stress) damage, microarray screenings showed stronger inductions by genotoxic carcinogens^37^. We hypothesize that in our assay, different modes of action result in a different role of p53. This hypothesis is supported by previous studies, as *p53*-deficient fibroblasts of mice have increased rates of genotoxic-carcinogen induced apoptosis and a *p53* heterozygous mouse model is relatively insensitive to non-genotoxic insults^51^,^52^,^53^. However, it has to be noted that Smed-p53 was reported to have both a tumor suppressor-like activity as well as a self-renewal function^54^. It is the first time a genetic knockdown is undertaken in the *in vivo* prediction and discrimination of carcinogens using invertebrates, and more compounds, concentrations and time points have to be tested in the future. This will be done in accordance with the approaches used in transgenic rodent gene mutation and other assays, and the dataset available via Kirkland *et al*.^55^. Even though the other tested knock-downs didn’t had a strong discriminative power, they can be useful to subdivide carcinogens based on underlying mechanisms of action.

**Figure 4:**
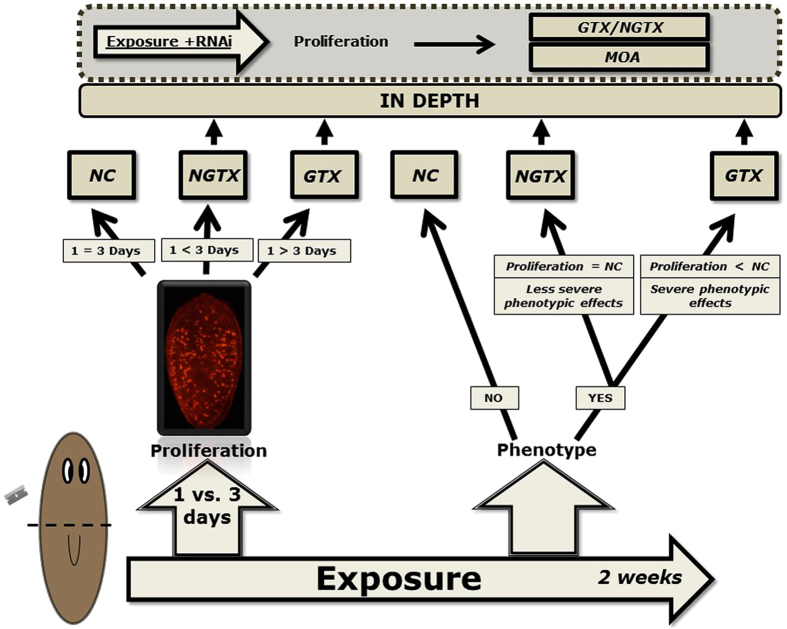
Workflow to predict and discriminate genotoxic (GTX), non-genotoxic (NGTX) and non-carcinogens (NC) based on *in vivo* proliferation responses of adult stem cells and phenotypic effects in the regenerating flatworm *S. mediterranea*. Step 1 of the workflow: measurement of the amount of dividing cells in a regenerating worm after 1 and 3 days of exposure (mitotic minimum and maximum during regeneration). GTX carcinogens: the amount of cells in mitosis is lower at 3 days as compared to 1 day of exposure (expressed relative to the non-exposed group) or is lower than 1. NGTX carcinogens: the amount of cells in mitosis is higher at 3 days of exposure as compared to 1 day of exposure (expressed relative to the non-exposed group) or is higher than 1. Non-carcinogens: the amount of cells in mitosis is the same at both days (expressed relative to the non-exposed group). Step 2 of the workflow: measurement of the amount of dividing cells at the moment of phenotypic appearance. A strong drop in cell proliferation is noticed when phenotype appears during gtx exposure. Step 3 of the workflow: when carcinogens are detected, a third step using knock donws can be included to increase sensitivity or to specify the mode of action of the compound.

Our work-flow pools previously discussed findings to enable a simple, rapid and inexpensive prediction and, for what is to our knowledge the first time, a discrimination of genotoxic and non-genotoxic carcinogens in an *in vivo* set-up (Fig. 4). By comparing proliferative responses of adult planarian stem cells during the initial stages of regeneration (i.e. after 1 and 3 days exposure), we were able to discriminate a set of genotoxic from non-genotoxic carcinogens based on (1) the initial proliferative inhibition induced by genotoxic compounds and (2) the proliferation-stimulating abilities of non-genotoxic carcinogens, which even exceed regeneration-induced peaks; in a proliferation-sensitive system. Stem cell proliferation at the appearance of phenotypic effects was added as an additional biological endpoint to the assay. A more sensitive animal, a subdivision of carcinogenic compounds according to common mechanisms of action, or a further confirmation of the genotoxic vs non-genotoxic classification can be achieved via appropriate knockdowns. For now, *p53* knockdowns discriminated both groups for the tested compounds, but future investigation on more compounds and other gene-knockdowns is needed and can be of added value in our workflow. To our opinion, the proposed concept is advantageous as it is a non-vertebrate, fast, sensitive and low-cost procedure, which can easily be applied universally and worldwide. It is very promising towards future risk assessments. We acknowledge that our proof of concept has to be further validated and valorized. Future focuses include a larger amount of compounds, in which inter-laboratory transferability, blind trials and regulatory actions are considered. It is essential for any new method that an approach towards international acceptance, government and industry is explored. Moreover, more compounds will render proper sensitivity and specificity values to compare our test with current alternatives such as the SHE assay, among others. Compound selection will be based on previous and current overviews^30^,^55^, and future strategies will be based on OECD test guidelines, among others^56^,^57^,^58^.

## Additional Information

**How to cite this article:** Stevens, A.-S. *et al*. Stem cell proliferation patterns as an alternative for *in vivo* prediction and discrimination of carcinogenic compounds. *Sci. Rep.* **7**, 45616; doi: 10.1038/srep45616 (2017).

**Publisher's note:** Springer Nature remains neutral with regard to jurisdictional claims in published maps and institutional affiliations.

## Supplementary Material

Supplemental Figures

## Figures and Tables

**Table 1:**
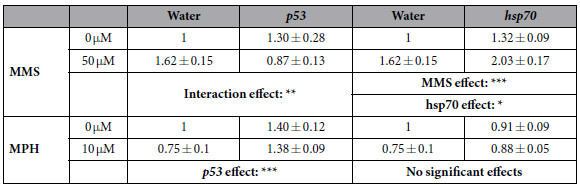
Stem cell divisions in response to genotoxic and non-genotoxic carcinogen exposures and in combination with a cancer-related gene knockdown.

Mitotic divisions per mm^2^ after 17 days exposure to a genotoxic (MMS) or non-genotoxic (MPH) carcinogen, with or without RNAi knockdown of *p53* or *hsp70*. The number of mitotic cells was normalized against the total body area of the worms and expressed relative to the corresponding non-exposed, non-RNAi group. The values indicated in the graphs are the average and standard error (se) of minimum 3 biological repeats. Significant carcinogen, RNAi and interaction effects are represented with corresponding p-values. Non-RNAi groups were injected with water. The average ± se of non-RNAi groups exposed to culture medium are: 166.0 ± 26.1 cells/mm^2^ (MMS) and 298.0 ± 34.1 cells/mm^2^ (MPH).

*p53* tumor protein 53, *hsp70* heat shock protein 70, *MMS* methyl methane sulphonate, *MPH* methapyrilene hydrochloride.

## References

[b1] EU. (ed Council Regulation (EC)) (Official Journal of the European Union, L 142/1—L 142/739, 2008).

[b2] Marone P. A., Hall W. C. & Hayes A. W. Reassessing the two-year rodent carcinogenicity bioassay: a review of the applicability to human risk and current perspectives. Regulatory toxicology and pharmacology: RTP 68, 108–118 (2014). 24287155 10.1016/j.yrtph.2013.11.011

[b3] Heinonen T., Louekari K. & Tähti H. Need for Harmonized Strategies and Improved Assessment of Carcinogenic and Genotoxic Potencies of Chemical Substances. Journal of Translational Toxicology 1, 76–87 (2014).

[b4] ICCVAM. ICCVAM Authorization Act of 2000. Public Law 106–545 (2000).

[b5] Doktorova T. Y., Pauwels M., Vinken M., Vanhaecke T. & Rogiers V. Opportunities for an alternative integrating testing strategy for carcinogen hazard assessment? Critical Reviews in Toxicology 42, 91–106 (2012). 22141324 10.3109/10408444.2011.623151

[b6] Silva Lima B. & Van der Laan J. W. Mechanisms of Nongenotoxic Carcinogenesis and Assessment of the Human Hazard. Regulatory Toxicology and Pharmacology 32, 135–143 (2000). 11067770 10.1006/rtph.2000.1427

[b7] Bogdanffy M. S. & Valentine R. Differentiating between local cytotoxicity, mitogenesis, and genotoxicity in carcinogen risk assessments: the case of vinyl acetate. Toxicology Letters 140–141, 83–98 (2003). 10.1016/s0378-4274(02)00504-0 12676454

[b8] Hernández L. G., van Steeg H., Luijten M. & van Benthem J. Mechanisms of non-genotoxic carcinogens and importance of a weight of evidence approach. Mutation Research/Reviews in Mutation Research 682, 94–109 (2009). 10.1016/j.mrrev.2009.07.002 19631282

[b9] van Delft J. H. . Discrimination of genotoxic from non-genotoxic carcinogens by gene expression profiling. Carcinogenesis 25, 1265–1276 (2004). 14963013 10.1093/carcin/bgh108

[b10] Kirkland D., Aardema M., Henderson L. & Müller L. Evaluation of the ability of a battery of three *in vitro* genotoxicity tests to discriminate rodent carcinogens and non-carcinogens: I. Sensitivity, specificity and relative predictivity. Mutation Research/Genetic Toxicology and Environmental Mutagenesis 584, 1–256 (2005). 10.1016/j.mrgentox.2005.02.004 15979392

[b11] Adler S. . Alternative (non-animal) methods for cosmetics testing: current status and future prospects-2010. Arch Toxicol 85, 367–485 (2011). 21533817 10.1007/s00204-011-0693-2

[b12] Benigni R., Bossa C. & Tcheremenskaia O. *In vitro* cell transformation assays for an integrated, alternative assessment of carcinogenicity: a data-based analysis. Mutagenesis 28, 107–116 (2013). 23132285 10.1093/mutage/ges059

[b13] Robb S. M. C., Ross E. & Alvarado A. S. SmedGD: the Schmidtea mediterranea genome database. Nucleic Acids Research 36, D599–D606 (2008). 17881371 10.1093/nar/gkm684 PMC2238899

[b14] Dunkel J., Talbot J. & Schotz E. M. Memory and obesity affect the population dynamics of asexual freshwater planarians. Physical biology 8, 026003 (2011). 21263170 10.1088/1478-3975/8/2/026003

[b15] Alexandrov V., Aiello C. & Rossi L. Modifying factors in prenatal carcinogenesis (review). In vivo (Athens, Greece) 4, 327–335 (1990). 2133106

[b16] Birnbaum L. S. & Fenton S. E. Cancer and developmental exposure to endocrine disruptors. Environ Health Perspect 111, 389–394 (2003). 12676588 10.1289/ehp.5686 PMC1241417

[b17] Stevens A. S. . Toxicity profiles and solvent-toxicant interference in the planarian Schmidtea mediterranea after dimethylsulfoxide (DMSO) exposure. J Appl Toxicol 35, 319–326 (2015). 24964768 10.1002/jat.3011

[b18] Stevens A. S. . Redox-Related Mechanisms to Rebalance Cancer-Deregulated Cell Growth The Anticarcinogenic Mechanisms of Regeneration. Curr Drug Targets 17, 1414–1437 (2016). 25944012 10.2174/1389450116666150506112817

[b19] Hanahan D. & Weinberg R. A. Hallmarks of Cancer: The Next Generation. Cell 144, 646–674 (2011). 21376230 10.1016/j.cell.2011.02.013

[b20] Best J. B. & Morita M. Planarians as a model system for *in vitro* teratogenesis studies. Teratogenesis, carcinogenesis, and mutagenesis 2, 277–291 (1982). 10.1002/1520-6866(1990)2:3/4<277::aid-tcm1770020309>3.0.co;2-8 6130627

[b21] Hall F., Morita M. & Best J. B. Neoplastic transformation in the planarian: I. Cocarcinogenesis and histopathology. The Journal of experimental zoology 240, 211–227 (1986). 3794621 10.1002/jez.1402400209

[b22] Schaeffer D. J. Planarians as a model system for *in vivo* tumorigenesis studies. Ecotoxicology and environmental safety 25, 1–18 (1993). 7682912 10.1006/eesa.1993.1001

[b23] Hall F., Morita M. & Best J. B. Neoplastic transformation in the planarian: II. Ultrastructure of malignant reticuloma. The Journal of experimental zoology 240, 229–244 (1986). 3794622 10.1002/jez.1402400210

[b24] Fu Z. & Tindall D. J. FOXOs, cancer and regulation of apoptosis. Oncogene 27, 2312–2319 (2008). 18391973 10.1038/onc.2008.24 PMC2819403

[b25] Benazzi M., Baguná J., Ballester R., Puccinelli I. & Papa R. D. Further Contribution to the Taxonomy of the ≪ Dugesia Lugubris-Polychroa Group ≫ with Description of Dugesia Mediterranea N.SP. (Tricladida, Paludicola). Bolletino di zoologia 42, 81–89 (1975).

[b26] Baguñà J. Estudios citotaxonómicos, ecológicos e histofisiología de la regulación morfogenética durante el crecimiento y la regeneración en la raza asexuada de la planaria Dugesia mediterranea. *Universitat de Barcelona, Barcelona* (1973).

[b27] Pirotte N. . Reactive Oxygen Species in Planarian Regeneration: An Upstream Necessity for Correct Patterning and Brain Formation. Oxid Med Cell Longev 2015, 392476 (2015). 26180588 10.1155/2015/392476 PMC4477255

[b28] Willems M. . An adult stem cell proliferation assay in the flatworm model *Macrostomum lignano* to predict the carcinogenicity of compounds. *Applied in vitro toxicology* Accepted (2015).

[b29] OECD O. f. E. C. a. D. Detailed review on cell transformation assays for detection of chemical carcinogens. *OECD Environment, Health and Safety Publications, Series on Testing and Assessment* No. 31 (2007).

[b30] Kirkland D., Kasper P., Müller L., Corvi R. & Speit G. Recommended lists of genotoxic and non-genotoxic chemicals for assessment of the performance of new or improved genotoxicity tests: A follow-up to an ECVAM workshop. Mutation Research/Genetic Toxicology and Environmental Mutagenesis 653, 99–108 (2008). 10.1016/j.mrgentox.2008.03.008 18539078

[b31] Robinson D. E. & Macdonald J. S. Background and framework for ILSI’s collaborative evaluation program on alternative models for carcinogenicity assessment. Toxicologic pathology 29, 13–19 (2001). 11695549 10.1080/019262301753178438

[b32] Saló E. & Baguñà J. Regeneration and pattern formation in planarians: I. The pattern of mitosis in anterior and posterior regeneration in Dugesia (G) tigrina, and a new proposal for blastema formation. Journal of Embryology and Experimental Morphology 83, 63–80 (1984). 6502076

[b33] Reddien P. W. & Alvarado A. S. FUNDAMENTALS OF PLANARIAN REGENERATION. Annual Review of Cell and Developmental Biology 20, 725–757 (2004). 10.1146/annurev.cellbio.20.010403.095114 15473858

[b34] Reddien P. W., Bermange A. L., Murfitt K. J., Jennings J. R. & Sanchez Alvarado A. Identification of genes needed for regeneration, stem cell function, and tissue homeostasis by systematic gene perturbation in planaria. Developmental cell 8, 635–649 (2005). 15866156 10.1016/j.devcel.2005.02.014 PMC2267917

[b35] Plusquin M. . Physiological and molecular characterisation of cadmium stress in Schmidtea mediterranea. The International journal of developmental biology 56, 183–191 (2012). 22451006 10.1387/ijdb.113485mp

[b36] Peiris T. H., Hoyer K. K. & Oviedo N. J. Innate immune system and tissue regeneration in planarians: An area ripe for exploration. *Seminars in immunology* (2014). 10.1016/j.smim.2014.06.005 PMC4171206 25082737

[b37] Ellinger-Ziegelbauer H., Stuart B., Wahle B., Bomann W. & Ahr H. J. Comparison of the expression profiles induced by genotoxic and nongenotoxic carcinogens in rat liver. Mutation research 575, 61–84 (2005). 15890375 10.1016/j.mrfmmm.2005.02.004

[b38] Oviedo N. J. & Beane W. S. Regeneration: The origin of cancer or a possible cure? Seminars in Cell & Developmental Biology 20, 557–564 (2009). 19427247 10.1016/j.semcdb.2009.04.005 PMC2706275

[b39] Riss J. . Cancers as Wounds that Do Not Heal: Differences and Similarities between Renal Regeneration/Repair and Renal Cell Carcinoma. Cancer research 66, 7216–7224 (2006). 16849569 10.1158/0008-5472.CAN-06-0040

[b40] Stevens A. S. . Redox-Related Mechanisms to Rebalance Cancer-Deregulated Cell Growth The Anticarcinogenic Mechanisms of Regeneration. *Curr Drug Targets* (2015). 10.2174/1389450116666150506112817 25944012

[b41] Hayes A. W. . A review of mammalian carcinogenicity study design and potential effects of alternate test procedures on the safety evaluation of food ingredients. Regulatory Toxicology and Pharmacology 60, S1–S34 (2011). 21094668 10.1016/j.yrtph.2010.10.002

[b42] Newmark P. A., Reddien P. W., Cebria F. & Alvarado A. S. Ingestion of bacterially expressed double-stranded RNA inhibits gene expression in planarians. Proceedings of the National Academy of Sciences 100, 11861–11865 (2003). 10.1073/pnas.1834205100 PMC304099 12917490

[b43] Josse R., Dumont J., Fautrel A., Robin M. A. & Guillouzo A. Identification of early target genes of aflatoxin B1 in human hepatocytes, inter-individual variability and comparison with other genotoxic compounds. Toxicology and applied pharmacology 258, 176–187 (2012). 22100608 10.1016/j.taap.2011.10.019

[b44] Ellinger-Ziegelbauer H., Stuart B., Wahle B., Bomann W. & Ahr H. J. Characteristic expression profiles induced by genotoxic carcinogens in rat liver. Toxicological sciences: an official journal of the Society of Toxicology 77, 19–34 (2004). 14600272 10.1093/toxsci/kfh016

[b45] Sionov R. V. & Haupt Y. The cellular response to p53: the decision between life and death. Oncogene 18, 6145–6157 (1999). 10557106 10.1038/sj.onc.1203130

[b46] Shirahige K. . Regulation of DNA-replication origins during cell-cycle progression. Nature 395, 618–621 (1998). 9783590 10.1038/27007

[b47] Hagstrom D., Cochet-Escartin O., Zhang S., Khuu C. & Collins E. M. Freshwater Planarians as an Alternative Animal Model for Neurotoxicology. Toxicological sciences: an official journal of the Society of Toxicology 147, 270–285 (2015). 26116028 10.1093/toxsci/kfv129 PMC4838007

[b48] Pagan O. R. . Planarians in pharmacology: parthenolide is a specific behavioral antagonist of cocaine in the planarian Girardia tigrina. The International journal of developmental biology 56, 193–196 (2012). 22451007 10.1387/ijdb.113486op

[b49] Melis J. P. M. . Detection of genotoxic and non-genotoxic carcinogens in Xpc−/−p53+/− mice. Toxicology and applied pharmacology 266, 289–297 (2013). 23153559 10.1016/j.taap.2012.11.004

[b50] Tennant R. W., French J. E. & Spalding J. W. Identifying chemical carcinogens and assessing potential risk in short-term bioassays using transgenic mouse models. Environmental health perspectives 103, 942–950 (1995). 8529591 10.1289/ehp.95103942 PMC1519166

[b51] Lackinger D., Eichhorn U. & Kaina B. Effect of ultraviolet light, methyl methanesulfonate and ionizing radiation on the genotoxic response and apoptosis of mouse fibroblasts lacking c-Fos, p53 or both. Mutagenesis 16, 233–241 (2001). 11320149 10.1093/mutage/16.3.233

[b52] Lips J. & Kaina B. DNA double-strand breaks trigger apoptosis in p53-deficient fibroblasts. Carcinogenesis 22, 579–585 (2001). 11285192 10.1093/carcin/22.4.579

[b53] Balachandra Dass S., Bucci T. J., Heflich R. H. & Casciano D. A. Evaluation of the transgenic p53+/− mouse for detecting genotoxic liver carcinogens in a short-term bioassay. Cancer Letters 143, 81–85 (1999). 10465341 10.1016/s0304-3835(99)00196-2

[b54] Pearson B. J. & Sanchez Alvarado A. A planarian p53 homolog regulates proliferation and self-renewal in adult stem cell lineages. Development 137, 213–221 (2010). 20040488 10.1242/dev.044297 PMC2799157

[b55] Kirkland D. J. . Updated recommended lists of genotoxic and non-genotoxic chemicals for assessment of the performance of new or improved genotoxicity tests. Mutat Res-Gen Tox En 795, 7–30 (2016). 10.1016/j.mrgentox.2015.10.006 26774663

[b56] Balls M. . Practical Aspects of the Validation of Toxicity Test Procedures. The Report and Recommendations of EVCAM Workshop. ATLA 23 (1995).

[b57] Edler L. & Ittrich C. Biostatistical Methods for the Validation of Alternative Methods for *In Vitro* Toxicity Testing. ATLA 31, 5–41 (2003). 15595899 10.1177/026119290303101s02

[b58] Dearfield K. L. . Next generation testing strategy for assessment of genomic damage: A conceptual framework and considerations. Environmental and Molecular Mutagenesis (2016). 10.1002/em.22045 27650663

