# Supplementary material for: Stem cell proliferation patterns as an alternative for in vivo prediction and discrimination of carcinogenic compounds
Source: Sci Rep. 2017 May 3;7:45616. doi: 10.1038/srep45616 (PMC5413882; doi:10.1038/srep45616)
Supplement: Supplemental Figures [file srep45616-s1.pdf]

# An alternative concept for *in vivo* prediction and discrimination of carcinogenic compounds using *Schmidtea mediterranea's* stem cell proliferation patterns

## *In vivo* prediction of carcinogens.

**An-Sofie Stevens, Maxime Willems, Michelle Plusquin, Jan-Pieter Ploem, Ellen Winckelmans, Tom Artois, Karen Smeets**

## Supporting information: supplemental figures and tables

**Figure S1. Stem cell divisions in response to genotoxic (GTX) carcinogens, non-genotoxic (NGTX) carcinogens and non-carcinogen (NC) exposures (1 and 2 weeks).** Mitotic divisions per $mm^2$ after 1 and 2 weeks exposure to a concentration range of genotoxic carcinogens (MMS and 4NQO), non-genotoxic carcinogens (MPH, CsA and CPZ) or non-carcinogens (Dmann). The number of mitotic cells was normalized against the total body area of the worms. The values indicated in the graphs are the average and standard error (se) of a minimum of 3 biological repeats. Significant effects as compared to control groups: ■ $p < 0.01$. 4NQO caused a general exposure effect after 2 weeks ($p < 0.01$). Control groups were exposed to culture medium or, for CsA experiments, to 0.05% DMSO (vehicle control). (abbreviations: *MMS* methyl methane sulphonate, *4NQO* 4-nitroquinoline -1-oxide, *MPH* methapyrilene hydrochloride, *CsA* cyclosporine A, *CPZ* chlorpromazine hydrochloride, *Dmann* D-mannitol)

| | Control | 1 week | | Control | 2 weeks | | |
|---|---|---|---|---|---|---|---|
| **MMS** | 347.5 ± 8.9 | **50 µM** 153.2 ± 16.0 | **200 µM** 32.1 ± 7.0 | 83.8 ± 25.9 | **20 µM** 135.7 ± 13.0 | **50 µM** 184.2 ± 17.6 | **100 µM** 139.1 ± 39.7 |
| **4NQO** | 211.9 ± 4.7 | **0.5 µM** 25.6 ± 10.0 | **2 µM** 1.2 ± 0.6 | 140.7 ± 14.1 | **0.05 µM** 193.2 ± 24.5 | **0.1 µM** 144.9 ± 52.1 | **0.2 µM** 33.5 ± 14.5 |
| **MPH** | 71.0 ± 13.5 | **10 µM** 104.1 ± 15.2 | **100 µM** 104.9 ±14.5 | 87.5 ± 39.0 | **10 µM** 113.0 ± 7.4 | **100 µM** 95.3 ± 23.1 | |
| **CsA** | 288.8 ± 17.7 | **0.25 µM** 279.4 ± 34.4 | **0.5 µM** 311.4 ± 25.7 | 180.9 ± 15.8 | **0.125 µM** 173.6 ± 17.5 | **0.25 µM** 173.3 ± 11.5 | **0.5 µM** 204.7 ± 29.9 |
| **CPZ** | 223.1 ± 24.8 | **1 µM** 195.3 ± 13.8 | **2 µM** 220.5 ± 27.0 | 136.3 ± 12.9 | **250 nM** 122.1 ± 14.4 | **500 nM** 181.4 ± 27.7 | **1 µM** 136.9 ± 33.0 |
| **Dmann** | 260.6 ± 44.0 | **16.5 mM** 274.5 ± 53.8 | **27.4 mM** 179.5 ± 43.8 | 135.9 ± 15.6 | **5.5 mM** 126.1 ± 13.4 | **16.5 mM** 129.3 ± 21.5 | **27.4 mM** 143.6 ± 18,2 |

**Figure S2. Images of stem cell proliferation patterns after 1 and 3 days of MMS exposure for each body part (head and tail).**

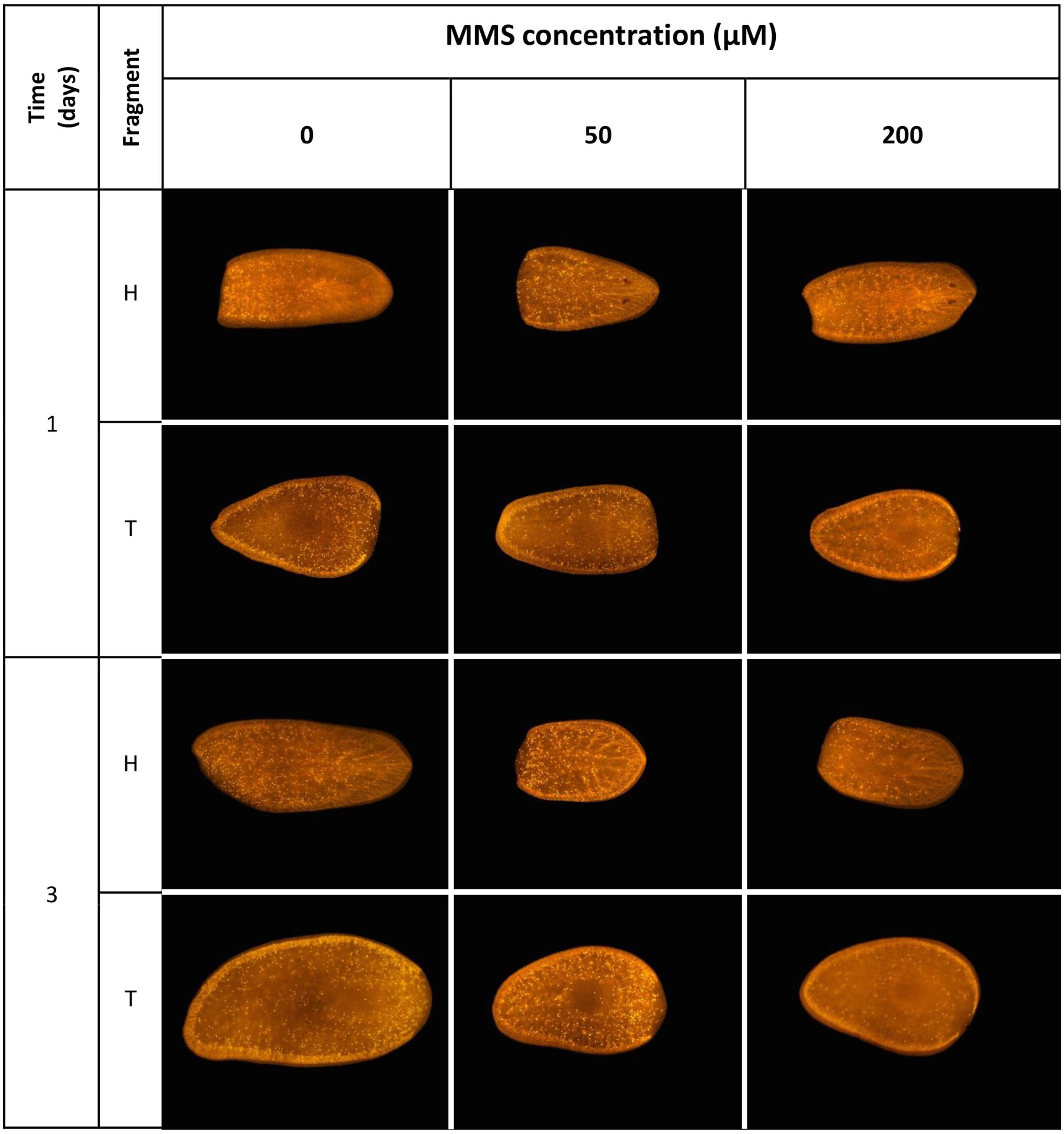

**Figure S3. Comparison of stem cell proliferation responses after 1 and 3 days of carcinogenic exposure for each body part (head and tail).** The figure represents mitotic divisions per $mm^2$ after 1 and 3 days (day=d) exposure to genotoxic carcinogens (MMS, 4NQO), non-genotoxic carcinogens (CsA, S-PB, MPH and CPZ) and non-carcinogens (Dmann) for head (B) and tail (C) body parts. Animals were cut above the pharynx. The number of mitotic cells was normalized against the total body area of the worms and expressed relative to the corresponding MMS, 4NQO, CsA, S-PB, MPH, CPZ and Dmann control group per time point, which was culture medium for all compounds except for CsA, where culture medium with 0.05% DMSO (vehicle control) was used. Cell proliferation values of each concentration are connected by lines and are the average and standard error (se) of minimum 3 biological repeats. Significant effects are indicated in the table: ***: p < 0.01; **: p < 0.05; *: p < 0. 1.

**A** Comparison of stem cell proliferation responses in heads and tails after 1 and 3 days of carcinogen exposure

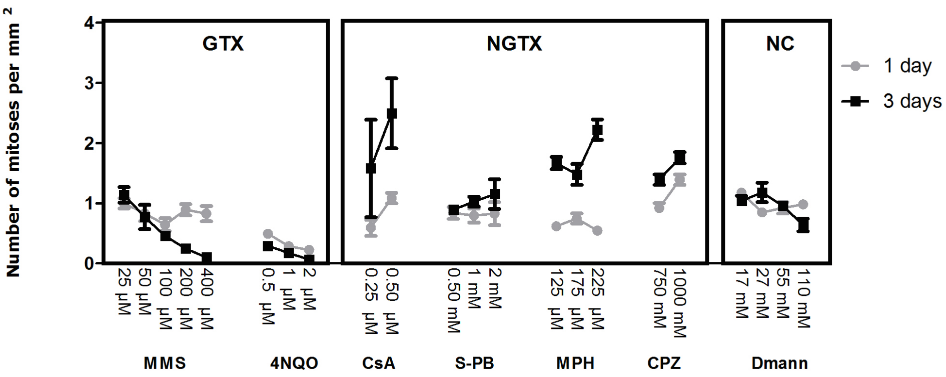


**B** Comparison of stem cell proliferation responses in heads after 1 and 3 days of carcinogen exposure

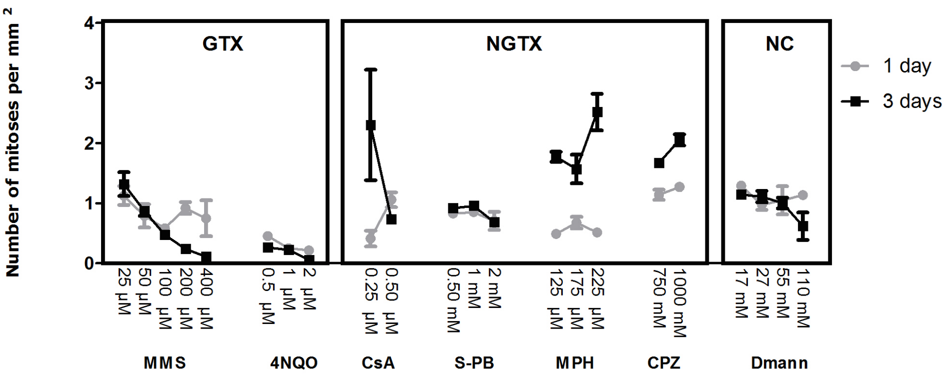


**C** Comparison of stem cell proliferation responses in tails after 1 and 3 days of carcinogen exposure

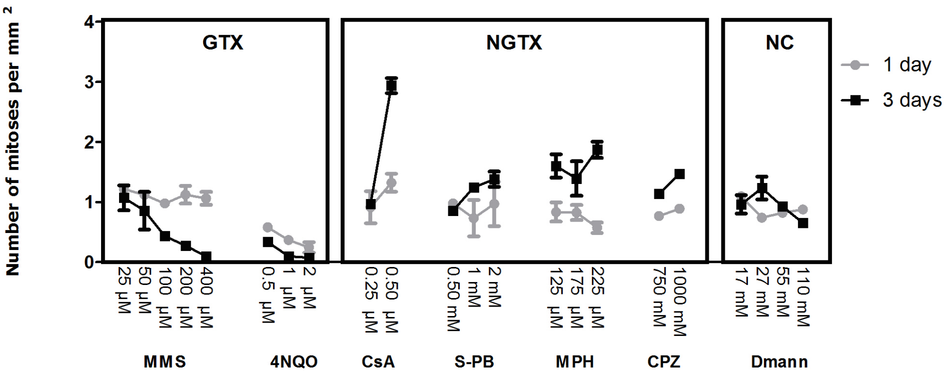

**Figure S4a. Classification of genotoxic (GTX) and non-genotoxic (NGTX) carcinogen concentrations according to observed phenotypes.** Concentrations of genotoxic (MMS, 4NQO) and non-genotoxic (S-PB, CPZ, MPH) carcinogens were classified into 3 categories according to the phenotypic effects they induced: Category 1: No effects on phenotype; Category 2: Phenotypic effects; Category 3: Mortality. For category 2 concentrations in bold, stem cell proliferation responses are presented in the article. Exposure times were 3 days (MMS), 1 week (4NQO) and 2 weeks (S-PB, CPZ, MPH). The phenotypes of these animals are represented in figure S4c. (abbreviations: *MMS* methyl methane sulphonate, *4NQO* 4-nitroquinoline-1-oxide, *S-PB* sodium phenobarbital, *CPZ* chlorpromazine hydrochloride, *MPH* methapyrilene hydrochloride)

| | | Toxicity | | |
|---|---|---|---|---|
| | | ***Cat. 1*** | ***Cat. 2*** | ***Cat. 3*** |
| **GTX** | **MMS** | 25-50-100-200 µM | 400-**500 µM** | 1 mM |
| | **4NQO** | 0.1-0.5 µM | **2 µM** | 5 µM |
| **NGTX** | **S-PB** | 1 mM | **2 mM** | 4 mM |
| | **CPZ** | 250-500 nM | 750-**1000 nM** | 2 µM |
| | **MPH** | 10 µM | **150**-250 µM | 500 µM |

**Figure S4b. Total number of animals with specific phenotypic effects after exposure to category 2 concentrations of genotoxic and non-genotoxic carcinogens.**

| | **GTX** | | **NGTX** | | |
|---|---|---|---|---|---|
| **Phenotypes** | MMS | 4NQO | S-PB | CPZ | MPH |
| *Tissue regression* | 9/26 | 5/10 | / | / | / |
| *Pigmentation effects* | 19/26 | 8/10 | / | / | / |
| *Behavioral effects* | 20/26 | 7/10 | 8/8 | 9/10 | 14/14 |
| *Regenerative failure* | 20/26 | 10/10 | / | / | / |
| *Effects on body size* | 22/26 | / | / | 5/8 | 12/14 |

**Figure S4c. Observed phenotypes in Cat. 2 animals.** The phenotypes of Cat. 2 animals as indicated in figure S4a are represented. In addition to the time points and concentrations indicated in figure S4a, a less severe phenotype of a lower MMS concentration is showed at a lower concentration. At the moment the first MMS phenotype appeared, this concentration was a Cat. 1 concentration. At a later time point (5 days), a less severe phenotype appears. Stem cell proliferation is also dropped in these animals.

**Figure S5. Comparison of different exposure set-ups.** Mitotic divisions per $mm^2$ after 1 week and 17 days exposure to 200 and 50 µM methyl methane sulphonate (MMS) respectively, in which a comparison was made between a continuous exposure with medium refreshment once or twice a week and an intermittent exposure (2 days exposure in MMS, 3 days recovery in culture medium). The number of mitotic cells was normalized against the total body area of the worms and expressed relative to the corresponding control group which was exposed to culture medium. The values indicated in the graphs are the average and standard errors of a minimum of 3 biological repeats. Significant effects as compared to control groups: dark grey $p < 0.01$; light grey $p < 0.05$.

| | Continuous exposure | | Intermittent exposure |
|---|---|---|---|
| | 1 x / week | 2 x / week | |
| **1 week exposure (200 µM MMS)** | 0.2 ± 0.0 | 0.1 ± 0.0 | 0.6 ± 0.1 |
| **17 days exposure (50 µM MMS)** | 1.7 ± 0.3 | 2.0 ± 0.3 | 1.1 ± 0.2 |

**Figure S6. 17 days exposure to 50 µM MMS.** Mitotic divisions per $mm^2$ after 17 days exposure to 50 µM methyl methane sulphonate (MMS) were assessed in different independent experiments. The number of mitotic cells was normalized against the total body area of the worms and compared to the control group which was exposed to culture medium. The values indicated in the graphs are the average and standard errors of a minimum of 3 biological repeats. Significant effects as compared to control groups: dark grey $p < 0.01$; light grey $p < 0.05$.

| | Control | 50 µM MMS |
|---|---|---|
| **Experiment A** | 148.7 ± 10.1 | 296.7 ± 42.6 |
| **Experiment B** | 253.6 ± 23.4 | 354.1 ± 14.3 |
| **Experiment C** | 159.5 ± 12.8 | 252.8 ± 45.3 |
| **Experiment D** | 107.6 ± 10.9 | 211.2 ± 7.4 |
| **Experiment E** | 190.4 ± 19.2 | 314.5 ± 17.7 |

**Figure S7. Primers used to generate RNAi-probes.** Sf = sense forward, sr = sense reverse, asf = anti sense forward, asr = anti sense reverse. *Tumor protein 53 (p53), heat shock protein 70 (hsp70).*

| Gene | | Sequence | Probe length (base pairs |
|---|---|---|---|
| ***p53*** | sf | GGATCCTAATACGACTCACTATAGGTCAAACCGATAAAATGGGAAA | 344 |
| | sr | CAAATTGACAGTTGACGGAAG | |
| | asf | TCAAACCGATAAAATGGGAAA | |
| | asr | GGATCCTAATACGACTCACTATAGGCAAATTGACAGTTGACGGAAG | |
| ***hsp70*** | sf | GGATCCTAATACGACTCACTATAGGGTTGTTGTTGGACGTTGCAC | 440 |
| | sr | CAGTTACCCGCTCTTTCTGC | |
| | asf | GTTGTTGTTGGACGTTGCAC | |
| | asr | GGATCCTAATACGACTCACTATAGGCAGTTACCCGCTCTTTCTGC | |
